# Clinico-biochemical factors to early predict biliary etiology of acute pancreatitis: age, female gender, and ALT

**Published:** 2015

**Authors:** NO Zarnescu, R Costea, EC Zarnescu (Vasiliu), S Neagu

**Affiliations:** * Second Department of Surgery, University Emergency Hospital Bucharest, Romania; ** “Carol Davila” University of Medicine and Pharmacy Bucharest, Romania

**Keywords:** acute pancreatitis, gallstone, biliary pancreatitis, risk score, biochemical prediction

## Abstract

**Background/ Aims:** Despite the existence of an easy tool to diagnose biliary tract disease as an etiology for acute pancreatitis (AP), the sensitivity of abdominal ultrasound is around 80%, which can be even lower in certain conditions.

**Methodology:** We have retrospectively reviewed data of 146 patients admitted for acute pancreatitis between 1999 and 2013. Bivariate analysis for clinical and biochemical variables was performed with respect to etiology of AP (biliary versus non-biliary). Multivariate analysis was performed by using binary logistic regression.

**Results:** There were 87 males (59.6%) and 59 females (40.4%), with a median age of 51. The etiology of acute pancreatitis was biliary in 71 patients (48.6%). Bivariate analysis found the following as significant association (p=0.001) with biliary pancreatitis: older age, female gender, and elevated AST, ALT. A binary logistic regression analysis identified as predictor factors for biliary etiology of acute pancreatitis: age OR = 1.031 (95% CI 1.004 - 1.059, p = 0.024), sex (female) OR = 2.34 (95% CI 1.022 - 5.359, p = 0.044) and ALT OR = 1.004 (95% CI 1.001 - 1.007, p =0.004). The two clinical scores included the three variables (A.S.ALT scores) in categorical format were generated and then checked with the ROC curves (areas under curve are 0.768 and 0.778).

**Conclusions:** Age, female gender, and elevated ALT can help identifying cases with biliary etiology of acute pancreatitis.

## Introduction

Gallstones represent more than one third of the etiology of acute pancreatitis [**1**]. Abdominal ultrasound (AUS) represents the standard tool of diagnosis for biliary tract disease with a high sensitivity and specificity around 95%. However, the sensitivity of AUS in the setting of acute pancreatitis is reduced (around 80%) due to overlying air-filled loops of bowel or small dimensions of stones [**2**]. It is important to note that the lack of biliary dilation on AUS does not exclude common bile duct stones as etiology of acute pancreatitis, especially in the first 48 hours from onset. The current best diagnostic tool (sensitivity of 98% and specificity of 99%) of choledocholithiasis is endoscopic ultrasound (EUS), but the availability is more reduced compared to ultrasound [**3**].

The potential more severe forms of acute pancreatitis can be explained by persistent obstruction of ampulla by a common bile duct stone [**4**]. For this reason, early ERCP with endoscopic sphincterotomy was initially suggested as a standard part of the treatment of acute biliary pancreatitis. However, the results of five prospective randomized clinical trials led to the current indications for the ERCP in patients with predicted severe acute pancreatitis: co-existing biliary obstruction or cholangitis [**5**-**10**]. On the other hand, patients with acute biliary pancreatitis that did not receive cholecystectomy (due to medical comorbidities or undiagnosed gallstones) are at high risk (around 30%) of recurrent acute pancreatitis [**11**]. In this context, the importance of properly identifying the biliary etiology is highlighted by one study that founded a higher mortality for recurrent cases of acute pancreatitis [**1**]. 

There are several reports describing clinical and/ or biochemical factors that can predict biliary etiology of acute pancreatitis, but some of them are difficult to use in daily practice [**2**,**12**-**15**]. The present paper describes our results by using data from 71 patients with gallstone etiology, out of 146 patients with acute pancreatitis. 

## Material and methods

We reviewed the medical charts of 146 patients who were admitted in our department between 1999 and 2013 with the diagnosis of acute pancreatitis. All data was retrospectively abstracted from de-identified patient records. Patients in whom a cholecystectomy was previously performed and post-ERCP pancreatitis patients were excluded from our study. Data for analysis included patient age, gender, obesity, clinical data (fever, onset-admission interval), and standard blood values. All biliary cases were confirmed by serial abdominal ultrasonography. The AUS was performed in the emergency room and later, after ward admission.

**Statistical analysis**

All values were presented as median and interquartile range (IQR) or proportions. Normality was assessed with Shapiro-Wilk test. Bivariate comparisons were assessed by using the Student T-Test (for parametric distribution) or Mann-Whitney U test (for non-parametric distribution) for continuous variables and either Chi-square or Fisher-exact test for categorical variables. Logistic binary regression analysis was used to assess factors associated with biliary etiology of acute pancreatitis. Potential variables for regression models were selected based on bivariate associations (p <0.10). After the identification of independent variables, we transformed the continuous variables in categorical ones (for an easier practical use) and created two clinico-biochemical scores that were assessed with ROC (receiver operating characteristic) curve. A two-sided p value of < 0.05 was considered significant. Statistical analyses were performed by using SPSS software package, version 15 (SPSS Inc, Chicago, IL).

## Results

There were 87 males (59.6%) and 59 females (40.4%), with a median age of 51 (IQR, 38-65). The etiology of acute pancreatitis was biliary in 71 patients (48.6%), alcohol in 33 patients (22.6%), other causes (hypertriglyceridemia, trauma, drugs) in 12 patients (8.2%) and idiopathic in 30 cases (20.6%). The interval between the onset of pancreatitis and hospital admission was under 24 hours in 47 patients (32.2%). 

The bivariate analysis of the categorical variables (**Table 1**) found that female and fever at admission, were statistically significant associated with biliary etiology. Previous attacks of acute pancreatitis were associated with non-biliary etiology.

**Table 1:** Bivariate analysis for categorical variables according to the etiology of acute pancreatitis

Variable	Non-biliary etiology (N=75)	Biliary etiology (N=71)	p-values
Female gender (N=59)	20 (26.7%)	39 (54.9%)	0.001*
Obesity (N=32)	12 (16%)	20 (28.2%)	0.11
Fever (N=21)	6 (8%)	15 (21.1%)	0.02*
Previous attacks of pancreatitis (N=32)	24 (32%)	8 (11.3%)	0.003*
Interval onset-admission < 24 hours (N=47)	19 (25.3%)	28 (39.4%)	0.34
*p<0.05			

Bivariate analysis of the continuous variables (**Table 2**) found as significant (p<0.05), associated with gallstone etiology of acute pancreatitis age and elevated liver enzymes (ALT and AST). The analysis showed a 10 years difference of age between the two groups and a three times elevation of both ALT and AST. There was a trend for higher total bilirubin level in the biliary patients, but without significance. There was no difference between groups regarding direct bilirubin or amylase level. 

**Table 2:** Bivariate analysis for continuous variables according to the etiology of acute pancreatitis

Variable	Non-biliary etiology (N=75)	Biliary etiology (N=71)	p-values
Age (years)	43 (35 - 59)	57 (47-68)	<0.001*
Total bilirubin (mg/ dL)	1.2 (0.8 - 1.8)	1.3 (0.9 – 2.9)	0.08
Direct bilirubin (mg/ dL)	0.5 (0.4 - 1.4)	0.6 (0.3 – 1.1)	0.85
AST (U/ L)	48.5 (31.2 - 80.2)	135 (78.3 – 298.3)	<0.001*
ALT (U/ L)	47 (28.5 - 76)	140 (77.8 – 327.3)	<0.001*
Amylase (U/ L)	498 (104 - 1300)	816 (207 - 1300)	0.13
WBC (/ mm)	12700 (10400 -18500)	12775 (9675-17150)	0.87
Hemoglobin (g/ dL)	13.8 (11.6 - 15.2)	13.8 (12.4 - 15)	0.81
BUN (mg/ dL)	38 (12.2 - 47.1)	26.7 (25 – 50.5)	0.88
Creatinine (mg/ dL)	0.8 (0.7 - 1.1)	0.9 (0.7 – 1.3)	0.55
Glucose (mg/ dL)	128 (105 - 167)	131 (102 - 172)	0.72
*p<0.05			

Our next aim was to define those factors independently predicting the biliary etiology of acute pancreatitis. Before performing the logistic regression, we tested the correlation between eligible continuous variables. There was a highly significant correlation between ALT and AST (r = 0.848, p<0.001). Because ALT was stronger associated with gallstone etiology of AP, AST was not included in further analysis. Based on our number of patients included in this study, the maximum number of variables to be assessed was four. For the multivariate logistic regression (**Table 3**) we used the following variables: gender, age, ALT and total bilirubin. Using a binary logistic regression model that incorporated these variables, our analysis demonstrated that age, sex and ALT level were the only independent factors associated with biliary etiology (p<0.05).

**Table 3:** Multivariable logistic regression of factors predicting the biliary etiology for acute pancreatitis

	p-values	OR (odds ratio)	95% CI (Confidence Interval)
Age	0.024*	1.031	1.004 - 1.059
Sex (female)	0.044*	2.34	1.022 - 5.359
ALT	0.004*	1.004	1.001 - 1.007
*p<0.05			

## Discussion

This analysis identified as independent predictors for biliary etiology of acute pancreatitis female gender, older age and elevated ALT. This study confirmed the result from two previous studies that identified the same variables [**12**,**13**]. 

Because gallstones are more common in female gender, one can explain the significant association between gender and etiology of acute pancreatitis. The most frequent cited biochemical modification associated with biliary etiology is represented by elevation of alanine aminotransferase (ALT), levels greater than threefold being suggestive for diagnosis [**12**,**13**,**16**]. Serum amylase levels greater than 1000 were postulated to suggest a biliary cause of acute pancreatitis [**17**]. Despite the fact that our analysis could not confirm this, one must consider the physiological temporal modifications of the amylase level in acute pancreatitis corroborated with the interval between the onset and hospital admission (in our study only one third of the patients came to hospital earlier than 24 hours from the onset of pancreatitis). The current study confirmed that level of total bilirubin is not useful predicting biliary etiology [**16**].

One prospective study that included 213 patients with acute pancreatitis (biliary etiology - 62%) calculated that the probability of a biliary origin of AP could be estimated by the following formula: = 1/1 + exp (4.6967 - 0.0656 x age + 1.1208 x sex - 0.6909 x ALT) [**13**]. However, such a formula is very difficult to use in clinical practice. Therefore, our goal was to generate an easy clinical tool that could be integrated in daily routine. For an easier use of the results from multivariate analysis, we transformed the continuous variables in categorical ones and we generated two clinico-biochemical scores (**Table 4**) for evaluation.

**Table 4:** A.S.ALT clinico-biochemical scores

	A.S.ALT score 1		A.S.ALT score 2	
	Variables	Points	Variables	Points
Age	Age < 50	0	Age < 40	0
	Age ≥ 50	1	Age 40 – 60	1
			Age ≥ 60	2
Sex	Male	0	Male	0
	Female	1	Female	1
ALT	ALT < 150	0	ALT < 50	0
	ALT ≥ 150	1	ALT 50 – 150	1
			ALT ≥ 150	2

**Fig. 1:**
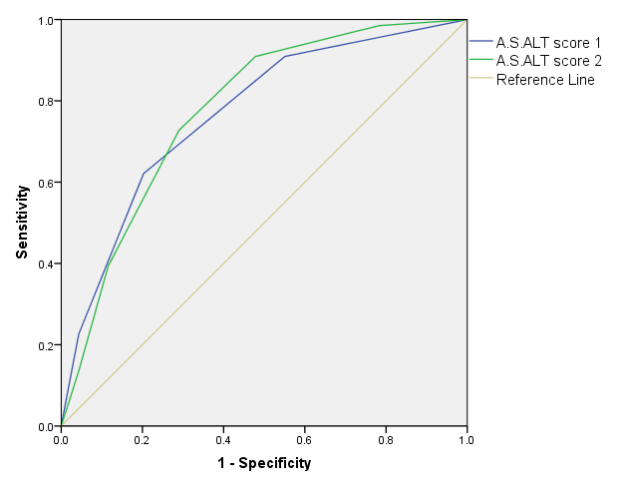
ROC curve of the A.S.ALT score predicting the biliary etiology of acute pancreatitis

In order to assess the clinical utility of these scores and identify the better one, we have further tested both A.S.ALT scores by using the ROC curve analysis (**Fig. 1**). 

The ROC analysis of both A.S.ALT scores found an area under curve (AUC) of 0.768 (95% CI 0.688 – 0.847) for the first score and AUC of 0.778 (95% CI 0.699 – 0.856) for the second one. Despite good results, further studies are needed in order to obtain a versatile highly performant clinico-biochemical score. 

There are two possible limitations for our study. First, due to the lack of echoendoscopy evaluation it is possible that some cases are actually gallstone-related pancreatitis and missed from the analysis. However, our data is relying on serial confirmatory abdominal echography, which increase the sensitivity of the test [**18**]. Second, because of the restrictive criteria of admission in a surgical clinic, the proportion of gallstones are higher compared to the whole group of patients with acute pancreatitis and the results might not be applicable in a lower incidence of biliary etiology setting such emergency room. 

Using a clinico-biochemical score might a very good tool for the patient’s selection for EUS final diagnosis of biliary acute pancreatitis. The importance of early detecting the biliary etiology of acute pancreatitis resides in the therapeutic implications related to the possibility of identifying all patients that might benefit from early ERCP with sphincterotomy and/ or cholecystectomy. The described three factors (**A**ge, **S**ex and **ALT**) are objective parameters and easy to obtain, but further studies are needed to confirm the present results. 

**Disclosures:**

None
